# Prognostic significance of clinical and pathological stages on locally advanced rectal carcinoma after neoadjuvant chemoradiotherapy

**DOI:** 10.1186/s13014-015-0425-5

**Published:** 2015-06-04

**Authors:** Bixiu Wen, Luning Zhang, Chengtao Wang, Rong Huang, Haihua Peng, Tian Zhang, Jun Dong, Weiwei Xiao, Zhifan Zeng, Mengzhong Liu, Yuanhong Gao

**Affiliations:** Department of Radiation Oncology, First Affiliated Hospital of Sun Yat-sen University, Guangzhou, 510080 China; Department of Radiation Oncology, State Key Laboratory of Oncology in South China, Sun Yat-sen University Cancer Center, Guangzhou, 510060 China

**Keywords:** Rectal carcinoma, Neoadjuvant chemoradiotherapy, Clinical stage, Pathological stage, Treatment outcome

## Abstract

**Objective:**

To investigate prognostic significance of clinical and pathological stages in patients with locally advanced rectal carcinoma treated with neoadjuvant chemoradiotherapy (neo-CRT) and total mesorectal excision.

**Patients and methods:**

210 patients with locally advanced rectal carcinoma (cT3-4 or cN+) treated with neo-CRT followed by total mesorectal excision. Treatment outcomes were compared according to clinical and pathological stage. Overall survival (OS), disease free survival (DFS) among patients with different clinical stage and pathological stage after neo-CRT.

**Results:**

The median follow-up time was 47 months (range, 14–98 months). Clinical T stage was associated with 5 year OS (*p* = 0.042) and 5 year DFS (*p* = 0.014) while clinical N stage was not associated with 5 year OS (*p* = 0.440), 5 year DFS (*p* = 0.711). Pathological T stage was associate with 5 year OS (*p* = 0.001) and 5 year DFS (*p* = 0.046); and N stage was associated with 5 year OS (*p* = 0.001), 5 year DFS (*p* = 0.002). The pathological stage was further classified into three groups: ypT0–2N0 in 91 patients (43.3 %), ypT3–4N0 in 69 patients (32.9 %) and ypT0–4N+ in 50 patients (23.8 %). While pathological stage (ypT0–2 vs ypT3–4N0 vs ypT0–4N+) was associated with 5 year OS (87.9 %, 75.5 %, 56.7 %, *p* = 0.000), 5 year DFS (74.5 %, 77.4 %, 50.5 %, *p* = 0.003). Multivariate analysis showed that ypN stage was an independent prognostic factor for patients 5 year DFS.

**Conclusions:**

Pathological stage is strongly associated with treatment outcomes in patients with locally advanced rectal carcinoma treated with neo-CRT followed by total mesorectal excision, which may be used as guidance for further individualized treatment.

Colorectal cancer is the 5th most common malignant neoplasm in morbidity and mortality in China according to Globocan 2012. Most majority of patients were diagnosed as locally advanced disease, among them 49.9 % were diagnosed as Duck B stage, and 33.9 % Duck C stage [1]. In colorectal cancer, the morbidity of rectal cancer accounts for more than 37 % [2].

The treatment strategy “neoadjuvant chemoradiotherapy (Neo-CRT) plus total mesorectal excision (TME) and six months adjuvant chemotherapy” has been recommended as the standard of care for patients with locally advanced rectal cancer by National Comprehensive Cancer Network (NCCN) since 2006 [3, 4]. Compared with postoperative chemoradiotherapy, preoperative chemoradiotherapy has been demonstrated to improve local tumor control, reduce treatment-related toxicity, and improve sphincter preservation [5–8]. The finding that postoperative pathological stage usually differs from pretreatment clinical stage complicates the ability to prognosticate outcome in patients treated with neoadjuvant therapy. It has been reported [9–11] that posttreatment pathological TNM stage is more closely related to the prognosis than pretreatment clinical stage in these patients. Patients with locally advanced rectal cancer may demonstrate a different treatment response to Neo-CRT ranging from pathological complete response (pCR) to resistance to Neo-CRT. Studies have reported that pathological TNM stage is of prognostic significance in treatment outcomes after Neo-CRT when compared to clinical TNM stage. Bujko *et al*. [12] reported that prognostic effect of adjuvant chemotherapy in patients with ypT0–4N0 disease after Neo-CRT and TME is not convincing, suggesting that adjuvant chemotherapy should be given individually according to pathological TNM stage.

The purpose of the study was to investigate treatment response in patients with locally advanced rectal cancer treated with Neo-CRT and TME and to compare prognostic effects of clinical and pathological TNM stages.

## Materials and methods

From March 2003 to December 2010, 210 patients with locally advanced rectal cancer (cT3–4 or cN+) were treated with Neo-CRT followed by TME with or without adjuvant chemotherapy at the Sun Yat-Sen University Cancer Center. Diagnosis was pathologically confirmed prior to Neo-CRT. Pretreatment clinical stage was determined by physical examination, ultrasound colonoscopy/colonoscopy, computerized tomography and/or magnetic resonance imaging.

In the present study, preoperative radiotherapy (RT) was performed with three-dimensional conformal radiation therapy (3D-CRT) technique with 6–8 MV X-ray and 3–4 fields at the full pelvis. The target volume definition was followed by the International Commission of Radiation Units 50 report recommendations. The delineation of clinical target volume (CTV) included primary rectal carcinoma, both ends of the affected rectum, the surrounding tissues of the affected rectum, the mesorectal area, the presacral lymph nodes, the obturator lymph nodes, and the iliac lymph nodes. For patients with stage T4 rectal carcinoma with bladder involvement, the delineation of CTV also included external iliac lymph nodes. The planned target volume (PTV) was designated as 8–10 mm margin from the CTV. For the dose prescription was as follows: 100 % of the prescription dose covered at least 95 % of the PTV; 95 % of the prescription dose covered at least 100 % of the PTV. The reference point was set as the intersection of the central axes of the three or four beams. The median radiation dose to PTV was 46.0 Gy (ranging from 30.0 to 50.0 Gy), 2 Gy per fraction, 5 fractions per week. The dose to the OARs was aimed to be as low as possible and must at least comply with the following constraints: bladder >50Gy in <50 % volume; Dmean of small bowel < 46Gy, small bowel > 50Gy in <5 % volume. Five patients who were operated on R1 or R2 resection were treated with postoperative radiotherapy with median dose of 36.0 Gy (range from 30.0 Gy to 40.0 Gy) delivered by 3D-CRT technique.

Two regimens of chemotherapy were administered during radiotherapy either the FOLFOX regimen (Fluorouracil, 3.0 g/m^2^, IV continuous infusion for 48 h on day 1; Leucovorin calcium, 200.0 mg/m^2^, IV bolus on day 1; Oxaliplatin, 100.0 mg/m^2^, IV on day 1; two cycles at an interval of 3 weeks), or the XELOX regiment (Capecitabine, 1000.0 mg/m^2^, on d1–14; Oxaliplatin, 100.0 mg/m^2^, IV on day 1; two cycles at an interval of 3 weeks). The postoperative adjuvant chemotherapy was either one of FOLFOX, XELOX or Capecitabine alone with median cycles of 2 (range from 2 to 6 cycles).

Radical surgery was performed according to the principles of total mesorectal excision. Postoperative pathological examination was performed according to the criteria developed by the AJCC/UICC (2002).

Treatment response including tumor regression response to Neo-CRT was investigated according to pathological TNM staging classification after radical surgery. Treatment outcome was analyzed according to clinical and pathological TNM stages in terms of overall survival (OS), disease free survival (DFS), local recurrence free survival (LRFS) and distant metastasis free survival (DMFS) among patients with different clinical stage (IIA, IIB, IIIA, IIIB and IIIC) and pathological stage (ypT0–2N0, ypT3–4N0 and ypT0–4N+).

All statistical analysis was performed using SPSS v17.0 software. *p* value of <0.05 was considered to be of statistical significance. *t*-test or the Wilcoxon rank sum test was used for continuous variables and the chi-square test for categorical variables. Kaplan-Meier analysis was performed for clinical outcome, and the log-rank test was used for comparison between clinical and pathological outcomes curves. Univariate and multivariate analysis of the prognostic factors were performed using Cox proportional hazard models.

## Results

### Patient characteristics

The patient characteristics are shown in Table 1. There were totally 210 patients including 149 males (71 %) and 61 females (29 %) with a median age of 56 years (range, 15–80 years). Patients with clinical stage IIA, IIB, IIIA, IIIB and IIIC were 31, 38, 4, 54 and 83, respectively. The median distance of carcinoma to the anus was 5 cm (1–16 cm). The median CEA level was 4.5 ng/mL (ranging from 0.2 ng/ml to 249.6 ng/mL). The tumor location of low-lying (distance between anal verge and lower edge of tumor ≤ 5 cm), middle-third (5 cm < distance between anal verge and lower edge of tumor ≤ 10 cm) and upper-third (distance btw anal verge and lower edge of tumor > 10 cm) patients were 130, 78 and 2, respectively.

**Table 1:** Baseline patient characteristics

Clinical characteristics	
Sex (*N*, %)	
Male	149 (71.0)
Female	61 (29.0)
Median age (years, range)	56 (15–80)
Median CEA level prior to treatment (μg/L, range)	4.5 (0.2–249.6)
Median hemoglobin level prior to treatment (g/L, range)	128 (64–170)
Tumor location (*N*, %)	
Low-lying	130 (61.9)
Middle-third	78 (37.1)
Upper-third	2 (1.0)
Median distance btw anal verge and lower edge of tumor (cm, range)	5.0 (1.0–16.0)
Clinical stage mordalities (*N*, %)	
Transrectal ultrasonograpy	187 (89.0)
CT	160 (76.2)
MRI	21 (10.0)
Clinical T stage (*N*, %)	
T1	2 (1.0)
T2	5 (2.4)
T3	79 (37.6)
T4	124 (59.0)
Clinical N stage (*N*, %)	
N0	69 (32.9)
N1	58 (27.6)
N2	83 (39.5)
Clinical TNM stage (*N*, %)	
IIA	31 (14.8)
IIB	38 (18.1)
IIIA	4 (1.9)
IIIB	54 (25.7)
IIIC	83 (39.5)
Concurrent chemotherapy regimen (*N*, %)	
FOLFOX	38 (18.1)
XELOX/Xeloda	164 (78.1)
Other regimen	8 (3.8)
Post-op adjuvant chemotherapy (*N*, %)	
Yes	155 (73.8)
None	55 (26.2)
Post-op adjuvant chemotherapy regimen (*N*, %)	
FOLFOX	13 (6.2)
XELOX/Xeloda	140 (66.7)
N/A	2 (1.0)

The median duration from the completion of radiotherapy to surgery was 42 days (interquartile range, 20–73 days). After Neo-CRT, 97.6 % (205/210 patients) of the patients were received R0 resection with Mile’s procedure in 102 patients (48.6 %), Dixon procedure in 104 patients (49.5 %) and Hartmann procedure in 4 patients (1.6 %). Sphincter preservation was obtained in 75 patients (42.2 %, 75/177 patients) who were assumed to receive Miles’ procedure during multidisciplinary evaluation. During the surgery, the median number of dissected lymph nodes was 6 (range, 0–37). The pathological stage was classified into ypT0–2N0 in 91 patients (43.3 %) with complete pathologic response in 52 patients (24.8 %), ypT3–4N0 in 69 patients (32.9 %) and ypT0–4N+ in 50 patients (23.8 %).

Five patients who were operated on R1 or R2 resection due to bladder/prostate involvement received postoperative radiotherapy with median dose of 36 Gy (range from 30 to 40 Gy) delivered by 3D-CRT technique. Among patients with ypT0–4N0 (160 patients), 115 received postoperative chemotherapy.

The complication after radical surgery included postponed incisions heal in 7 patients (3.3 %), wound infection in 2 patients (1.0 %), anastomotic leak in 2 patients (1.0 %) and pelvic sepsis in 1 patient. All of them were healed with best supportive care. The artificial anus was unobstructed.

### Patient outcome after Neo-CRT

The median follow-up was 47 months (range, 14–98 months). The 5 year OS and DFS for the whole group were 77.1 % and 70.4 %, respectively. The rates of local tumor recurrence and distant metastasis at 5 year were 7.1 % and 20.0 %, respectively. In the present study, there were 155 patients received post-op adjuvant chemotherapy, 55 patients did not. The 5 year OS, DFS, LRFS and DMFS for these two groups were 79.8 % and 69.2 % (*p* = 0.085), 73.5 % and 60.6 % (*p* = 0.072), 91.5 % and 89.5 % (*p* = 0.629) and 82.8 % and 71.2 % (*p* = 0.106), respectively. The 5 year LRFS in patients with low-lying, middle-third and upper-third rectal carcinoma was 87.1 %, 97.3 % and 100 %, respectively (*p* = 0.039).

Eleven patients developed single locoregional tumor recurrence that occurred at the first 3 years; 2 developed locoregional tumor recurrence and distant metastasis in the lung or bone; 29 patients developed distant metastasis with 14 in the lung, 11 in the liver, 2 in the bone, other 2 in the the abdominal or supraclavicular lymphadenopathy. Among them, 5 showed recurrence at the primary site, 4 at the iliac, 2 at the perirectal region, one at the primary site with lung metastasis and the other one at the iliac with lung and bone metastases. Twenty-nine patients showed distant metastasis alone after neo-CRT and TME. Among them, 14 patients showed distant metastasis at the lung, 11 at the liver, 2 at the bone, and 2 at lymph nodes at other site.

Five patients who were treated with Neo-CRT of 46 Gy and operated on R1 or R2 resection due to bladder/prostate involvement received postoperative radiotherapy with median dose of 36 Gy. Two of them died of the disease and the other 3 remains disease free survival.

At the end of the study, 43 patients had died (20.4 %) of recurrent or metastatic disease in the lung, liver and locoregional recurrence.

### Comparison of change between clinical and pathological stages after Neo-CRT

After Neo-CRT, postoperative pathological evaluation according to TNM classification in comparison with clinical stage showed that T stage decreased in 153 patients (72.9 %), and increased in 5 (2.4 %), and remained unchanged in 52 (24.8 %); N stage decreased in 116 patients (55.2 %), increased in 13 patients (6.2 %), remained unchanged in 20 patients with clinically positive lymph nodes (9.5 %) and 61 (29.0 %) with clinically negative lymph nodes.

After Neo-CRT, postoperative pathological evaluation according to TNM classification showed T downstage in 153 patients (72.9 %), N downstage in 116 patients (55.2 %) and pathological TNM downstage in 132 patients (62.9 %).

### Correlation of clinical stage with patient outcome

**Fig. 1:**
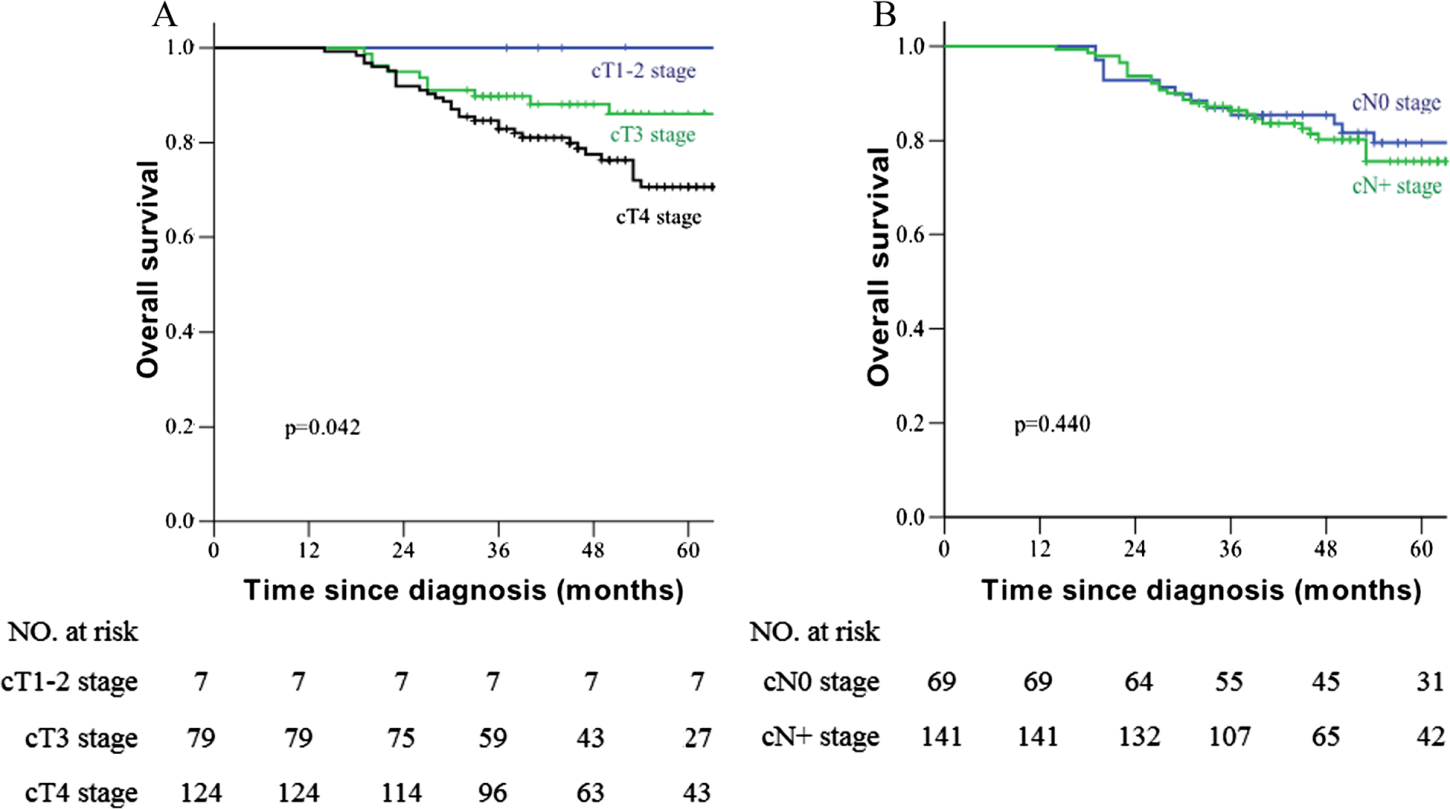
Kaplan-Meier overall survival **a, b** stratified by clinical T and N stage in patients with locally advanced rectal carcinoma who were treated with neo-adjuvant chemoradiotherapy and total mesorectal excision

In regards to the prognosis of patients with different clinical T stages in Fig. 1, the 5 year OS was 100 %, 86.1 %, and 70.7 % in patients with cT1–2, cT3, and cT4 rectal carcinoma, respectively (*p* = 0.042); the 5 year DFS was 100 %, 79.7 % and 68.7 % in patients with cT1–2, cT3, and cT4 rectal carcinoma, respectively (*p* = 0.014). The 5 year OS was 79.5 % and 75.6 % in patients with stage cN0 and cN+ rectal carcinoma, respectively (*p* = 0.440); the 5 year DFS was 66.2 % and 69.2 % in patients with stage cN0 and cN+ rectal carcinoma, respectively (*p* = 0.711).

For patients with clinical IIA, IIB, IIIA, IIIB and IIIC rectal carcinoma, the 5 year OS was 80.1 %, 79.0 %, 100.0 %, 74.4 % and 74.6 %, respectively (*p* = 0.661). The 5 year DFS was 74.2 %, 69.5 %, 100.0 %, 69.2 %, 66.2 %, respectfully (*p* = 0.662). The 5 year LRFS was 93.5 %, 86.6 %, 100.0 %, 96.2 % and 88.6 %, respectively (*p* = 0.361). The 5 year DMFS was 80.5 %, 86.5 %, 100.0 %, 72.5 % and 78.8 %, respectively (*p* = 0.241).

### Correlation of pathological stage with patient outcome

**Fig. 2:**
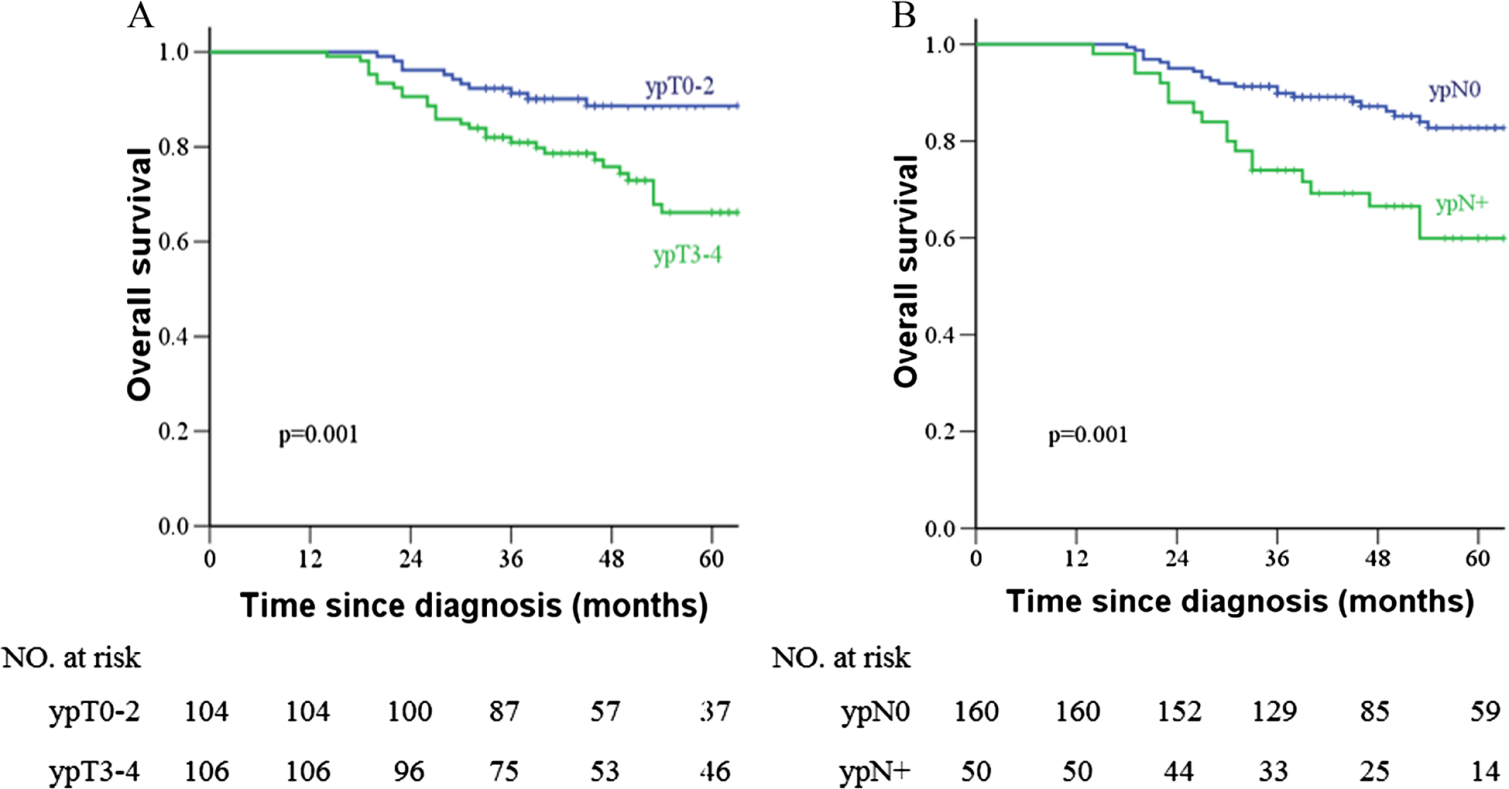
Kaplan-Meier overall survival **a, b** stratified by pathological T and N stage in patients with locally advanced rectal carcinoma who were treated with neo-adjuvant chemoradiotherapy and total mesorectal excision

As shown in Fig. 2 for the prognosis of patients with different pathological TN stages, the 5 year OS was 88.6 % and 66.2 % in patients with ypT0–2 and ypT3–4 rectal carcinoma, respectively (*p* = 0.001); the 5 year DFS was 75.0 % and 65.6 % in patients with ypT0–2 and ypT3–4 rectal carcinoma, respectively (*p* = 0.002). The 5 year OS was 82.8 % and 59.9 % in patients with ypN0 and ypN+ rectal carcinoma, respectively (*p* = 0.001); the 5 year DFS was 76.7 % and 54.1 % in patients with ypN0 and ypN+ rectal carcinoma, respectively (*p* = 0.002).

**Fig. 3:**
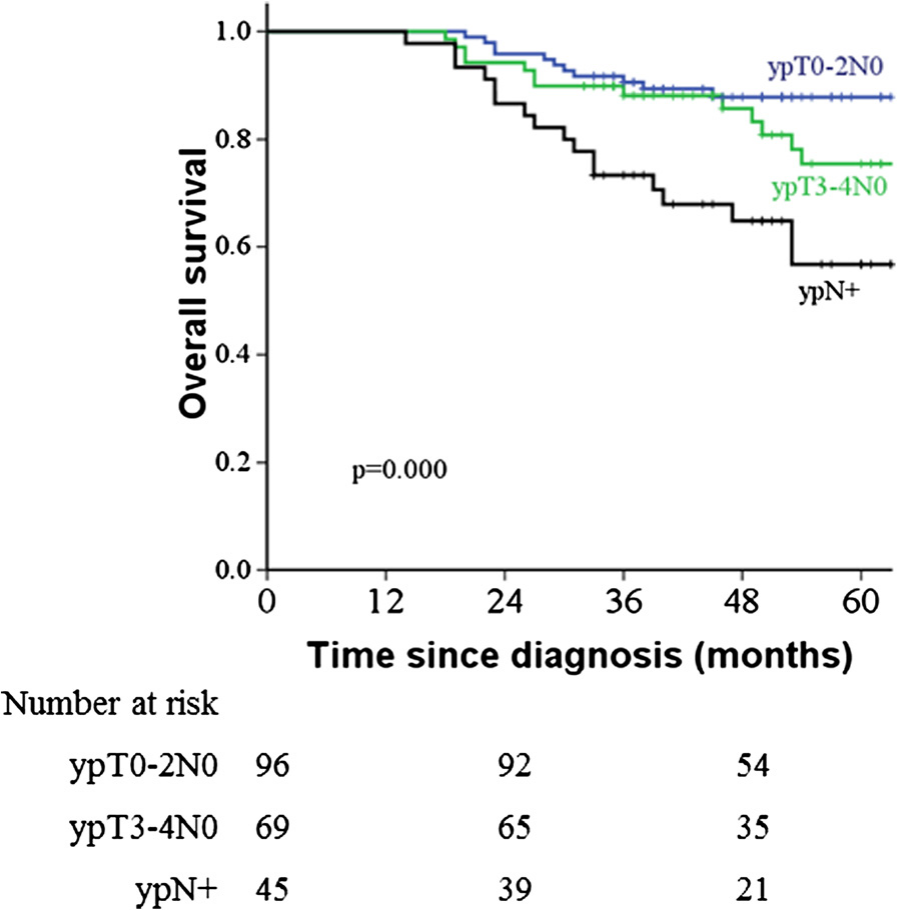
Kaplan-Meier overall survival stratified by pathological TN stage in patients with locally advanced rectal carcinoma who were treated with neo-adjuvant chemoradiotherapy and total mesorectal excision

The pathological stage was further classified into three groups: ypT0–2N0 (91 patients, 43.3 %), ypT3–4N0 (69 patients, 32.9 %) and ypT0-4N+ (50 patients, 23.8 %). The 5 year OS for groups 5 year OS for groups of ypT0–2N0, ypT3–4N0 and ypT0–4N+ was 87.9 %, 75.5 % and 56.7 % (*p* = 0.000), respectively; the 5 year DFS was 74.5 %, 77.4 % and 50.5 % (*p* = 0.003), respectively. In regards to the prognosis of patients with ypT0–2 and ypT3–4 rectal carcinoma, as shown in Fig. 3, the 5 year OS for patients with was 88.7 % and 66.1 % (*p* = 0.001), respectively; and the 5 year DFS was 75.5 % and 65.6 %, respectively (*p* = 0.046).

### Univariate analysis and multivariate analysis of prognostic factors for patients with locally advanced rectal carcinoma

Univariate analysis (Table 2) showed that pT, pN and pathological stages were associated with OS, DFS, LRFS and DMFS after Neo-CRT for patients with locally advanced rectal carcinoma. Clinical T stage was associated with DFS and distance between anal verge and lower edge of tumor was associated with LRFS. Multivariate analysis (Table 3) showed that only ypN stage was an independent prognostic factor for OS, DFS, LRFS and DMFS; ypT was associated with OS and DMFS. Patients with pN+ disease showed a high recurrence rate than those with pN0 disease (HR: 2.239;95 % CI:1.286–3.898). Pathological stage was independent prognostic factors for OS and DMFS.

**Table 2:** Univariate analysis of prognostic factors for patients with locally advanced rectal carcinoma

Variable	OS			DFS			LRFS			DMFS		
	HR	*p* value	95 % CI	HR	*p* value	95 % CI	HR	*p* value	95 % CI	HR	*p* value	95 % CI
Pathological stage												
ypT0–2N0	1			1			1			1		
ypT3–4N0	2.076	0.074	0.933–4.622	1.031	0.925	0.547–1.942	1.279	0.508	0.617–2.651	1.519	0.317	0.670–3.444
ypT0–4N+	3.688	0.001	1.702–7.911	2.255	0.007	1.246–4.081	2.816	0.003	1.429–5.546	3.429	0.002	1.602–7.339
pT stage												
ypT3–4 *vs*. ypT0–2	3.131	0.001	1.578–6.212	1.675	0.049	1.001–2.801	1.970	0.025	1.090–3.561	2.615	0.005	1.328–5.150
pN stage												
ypN+ *vs.* ypN0	2.595	0.002	1.414–4.760	2.252	0.004	1.333–3.804	2.519	0.002	1.411–4.498	2.889	0.001	1.540–5.420
Sex												
Female *vs.* Male	0.950	0.879	0.488–1.850	0.943	0.838	0.539–1.652	0.894	0.730	0.473–1.690	1.060	0.867	0.538–2.087
Age, years												
>56 *vs.* ≤56	1.243	0.477	0.682–2.266	1.243	0.396	0.752–2.056	1.433	0.215	0.812–2.532	1.444	0.248	0.774–2.695
Pretreatment CEA level, μg/L												
≥5.0 *vs.* <5.0	0.868	0.648	0.474–1.592	0.941	0.816	0.566–1.565	1.145	0.640	0.650–2.018	1.282	0.434	0.689–2.385
Pretreatment Hb level, g/L												
Anemia *vs.* normal	1.092	0.815	0.521–2.289	1.047	0.888	0.554–1.976	1.225	0.558	0.622–2.412	1.020	0.960	0.469–2.220
Distance btw anal verge and lower edge of tumor, cm												
≤5.0 *vs.* >5.0	1.294	0.429	0.683–2.449	1.643	0.082	0.939–2.877	5.446	0.023	1.257–23.586	0.907	0.761	0.481–1.707
Differentiation of tumor												
Well–*vs.* poorly differentiated	1.315	0.126	0.926–1.869	1.198	0.246	0.883–1.624	1.124	0.519	0.788–1.601	1.198	0.345	0.823–1.744
cT stage												
cT4 *vs*. cT1–3	1.837	0.074	0.943–3.577	1.856	0.030	1.060–3.249	1.735	0.083	0.931–3.233	1.648	0.148	0.838–3.241
cN stage												
cN+ *vs*. cN0	1.291	0.444	0.671–2.484	1.107	0.712	0.646–1.897	1.150	0.654	0.624–2.121	1.473	0.278	0.732–2.966
Post–op adjuvant chemotherapy												
Yes *vs.* None	1.712	0.089	0.921–3.181	1.614	0.074	0.955–2.726	0.792	0.122	0.589–1.065	1.687	0.110	0.888–3.207

**Table 3:** Multivariate analysis of prognostic factors for patients with locally advanced rectal carcinoma

Variable	OS			DFS			LRFS			DMFS		
	HR	*p* value	95 % CI	HR	*p* value	95 % CI	HR	*p* value	95 % CI	HR	*p* value	95 % CI
pN stage												
ypN+ *vs.* ypN0	2.206	0.016	1.159–4.196	2.239	0.004	1.286–3.898	2.239	0.003	1.286–3.898	2.678	0.004	1.374–5.218
pT stage												
ypT3–4 *vs*. ypT0–2	2.640	0.007	1.308–5.331	1.441	0.175	0.850–2.443	1.621	0.121	0.880–2.987	2.196	0.027	1.096–4.400
Sex												
Female *vs.* Male	0.859	0.663	0.433–1.702	0.861	0.605	0.487–1.522	0.794	0.486	0.414–1.520	0.924	0.824	0.461–1.853
Age, years												
>56 *vs.* ≤56	1.382	0.298	0.752–2.539	1.389	0.208	0.833–2.315	1.679	0.081	0.939–3.004	1.683	0.107	0.894>–3.167

## Discussion

Our study has demonstrated that neoadjuvant chemoradiotherapy is associated with tumour downstage with complete pathologic response rate of 24.8 %, elevated radical resection rate, sphincter preservation and no increase in surgical complication after total mesorectal excision in patients with locally advanced rectal carcinoma. Pathological TNM stage is strongly associated with treatment outcome in these patients after neo-CRT and may be of better prognostic significance than clinical TNM in terms of OS, DFS. Distant metastasis remains the main obstacle of the successful treatment for patients with locally advanced rectal carcinoma.

In recent years, studies have been conducted to investigate the influence of the postoperative pathological stage on the prognosis of locally advanced rectal carcinoma. Quah *et al.* [11] found that the postoperative pathological stage was related to the prognosis of 331 patients with locally advanced rectal carcinoma receiving neoadjuvant chemotherapy. In this study, pathological stage (ypT1–2N0, ypT3–4N0, and ypN+) was significantly correlated with DFS (*P* = 0.003) and OS (*P* = 0.000), and patients with stage ypT0N0 or ypT1–2N0 rectal carcinoma had a better prognosis. Park *et al.* [13] reported that in patients with stage ypT0N0, ypT1–2N0, and ypT3–4/N+ rectal carcinoma, the 5 year OS was 93.4 %, 87.0 %, and 77.3 % (*P* = 0.002), respectively. The 5 year DFS was 90.5 %, 78.7 %, and 58.5 %, respectively (*p* < 0.001) in 725 patients with locally advanced rectal carcinoma, and OS and DFS were significantly different among these patients.

Thus, these investigators proposed that the pathological stage after neoadjuvant chemotherapy was an important predictor, and recommended postoperative adjunctive therapeutics according to the response of rectal carcinoma patients to chemoradiotherapy, which was also known as individualized therapy. For example, a wait-and-see strategy can be applied in patients with stage ypT0N0 rectal carcinoma; intensified adjunctive chemotherapy is employed in patients with stage ypT3–4/N+ colorectal carcinoma. Kim *et al.* [14] reported that the 5 year DFS was 65.2 % and 35.7 % in patients with stage N0 and N+ rectal carcinoma, respectively, among 114 patients with locally advanced rectal carcinoma (*p* = 0.002), suggesting that pathological N stage is an important predictor [15].

Our results in univariate and multivariate analyses of prognostic factors also suggest that the pathological stage after neoadjuvant chemoradiotherapy significantly correlates with the prognosis of rectal carcinoma. In patients with ypT0–2N0, ypT3–4N0, and ypT0–4N+ rectal carcinoma, the 5 year OS was 87.9 %, 75.5 % and 56.7 % (*p* = 0.000), respectively; and the 5 year DFS was 74.5 %, 77.4 % and 50.5 % (*p* = 0.003). Stages ypT and ypN are important predictors for disease-free survival. Clinical TNM stage was not related to overall survival and distant metastatic free survival although cT stage was associated only with disease free survival in univariate analysis. Thus, our findings together with previously reported findings indicate that the postoperative pathological stages reflect the prognosis of patients with locally advanced rectal carcinoma better than does clinical stage before treatment, and it is more rational to choose and administer individualized adjuvant therapy according to the postoperative pathological stages [15].

Since pelvic lymph node status after neoadjuvant chemoradiotherapy is an important prognostic factor in patients with locally advanced rectal carcinoma, Jwa *et al.* [16] developed nomogram to improve prediction of lymph node status after neo-CRT by analyzing pretreatment ypT stage, patient age, tumor differentiation and clinical N stage before Neo-CRT, lymphovascular invasion and perineural invasion.

Stratified adjuvant therapy, performed according to pathological stage, has been reported in patients with locally advanced rectal carcinoma after neoadjuvant chemotherapy. Focal dissection can be performed in patients with locally advanced rectal carcinoma achieving pathological complete response (pCR) after neoadjuvant chemotherapy, and the therapeutic efficacy is similar to that after radical surgery [17, 18]. Moreover, this also avoids the complications and sequelae of radical surgery, and significantly improves quality of life. Govindarajan *et al.* [19] found that postoperative adjunctive chemotherapy had no influence on the 5 year DFS in patients with locally advanced colorectal carcinoma at pathological stage ypT0–2N0 after preoperative chemoradiotherapy. Thus, the value of postoperative adjunctive chemotherapy should be further investigated in future randomizedcontrolled trials.

In the present study, we found that the major cause of treatment failure in patients with locally advanced rectal carcinoma was distant metastasis. Patients with stage ypT3–4N0 and ypT0–4N+ rectal carcinoma had the highest rate of distant metastasis (35.7 %), which was markedly greater than that of patients with stage ypT0–2N0 rectal carcinoma (*P* < 0.001). Thus, an effective way to improve therapeutic efficacy is to reduce distant metastasis. For patients with rectal carcinoma at stage ypT3–4N0 or stage ypT0–4N+, postoperative adjunctive therapy should be changed to prolong the duration of treatment or increase the dose of chemotherapeutics, or to apply targeted therapy, which may reduce the rate of distant metastasis and improve the survival rate.

Similar to previous studies [11], we found that clinical stage before treatment failed to reflect the prognosis of patients with locally advanced rectal carcinoma. These were several underlying reasons. First, the pretreatment evaluation of clinical stage is different in different studies. The accuracy and specificity of the depth of colorectal carcinoma invasion (T stages) for endoscopic ultrasonography (EUS) are 94 % and 86 %, respectively [20, 21]; and for MRI 94 % and 69 %, respectively [22–25]. The sensitivity and specificity of determining the relationship between carcinoma and surrounding tissues for EUS are 90 % and 75 %, respectively; for MRI 82 % and 76 %, respectively; and for CT 55 % and 74 %, respectively [20, 26]. Thus, T stage and N stage may not accurately reflect the disease condition. Second, after combined use of chemotherapy and radiotherapy, additional factors may influence the response to treatment, such as carcinoma size, dose of radiation and chemotherapeutic regimen [27]. In addition, some molecular biological markers (such as epidermal growth factor receptor, thymidylate synthase, p21, and CEA) may also influence the response of colorectal carcinoma to chemoradiotherapy [28, 29].

Neoadjuvant chemoradiotherapy may reduce the pathological stage of rectal carcinoma to a different extent in different patients. In the present study, postoperative pathology confirmed that the pathological stage of rectal carcinoma was decreased in 132 patients (62.9 %) after neoadjuvant chemoradiotherapy, of whom patients with rectal carcinoma at stage ypT0–2N0 accounted for 43.3 %. This finding is consistent with previous reports [13, 30–32]. Patients with rectal carcinoma at stage ypT0–2N0 had a better prognosis.

In conclusion, neo-CRT and total mesorectal excision is an effective treatment modality for patients with locally advanced rectal carcinoma. The pathological stage is an important prognostic factor, which may be used as guidance for further individualized adjuvant therapy. Distant metastasis remains obstacle for the successful treatment of the disease. Further investigation therapeutic options are still needed to improve clinical outcomes of locally advanced rectal carcinoma.
